# Female Physicians

**Published:** 1848-01

**Authors:** 


### EDITORIAL DEPARTMENT.

---

*Female Physicians.*—The novel event of a female medical student being in attendance at the Geneva College having been the subject of remarks in Medical and other Periodicals, we are led to state the circumstances, which are briefly as folllows:—

Shortly after the commencement of the lecture session now in progress, the Faculty of Geneva College received an application, by letter, for the admission of a lady to the privileges of the Institution. The Faculty resolved to submit the letter (written by the applicant herself) to the class, and to return a favorable reply provided no objections thereto were entertained. The class adopted unanimously, resolutions expressing their willingness that the applicant should be received, and pledging themselves to treat her with respectful consideration. She was accordingly admitted, and has thus far attended the lectures in all the departments, as well as surgical operations, and dissections, personally participating in the latter. Nothing has transpired, as yet, to disprove the propriety of the action taken by the Faculty and class. In so far as her presence in the lecture room has had any influence, it has been conducive to a more strict observance of decorum than is usual with medical classes, and any embarrassment which may have been felt by all parties has long since disappeared.

It is understood that previously to her application to the Geneva College, she had applied to be received at the Institutions of Philadelphia, New-York city, and Boston, but without success. Geneva College will therefore be entitled to the distinction, meritorious or otherwise, of first practically exemplifying the experiment of opening the door of medical instruction to a female candidate for the medical profession.

Several inquiries suggest themselves in connection with the foregoing event. Is it likely that the example of Miss B. will be followed by other females? We think it by no means impossible that this will be the case. And we venture to predict, that, before many years, there will be far less difficulty than was experienced in the present instance, in finding a Medical Institution willing to entertain favorably similar applications, and that the fact of females qualifying themselves to become medical practitioners will have ceased to possess novelty. That females will become general practitioners is hardly to be expected, but that they may devote themselves successfully and usefully to special branches is not to be doubted. This has been sufficiently demonstrated in the instance of Mad. Boivin, and others, who have been distinguished not only as practitioners, but as writers and teachers. We should regret to see any innovations tending to lead woman from her approprite sphere—the domestic hearth, and the social circle; but we can perceive no good reasons why females whose duties are not prescribed by domestic or social relations, should be denied the privilege of obtaining a support, and of exercising usefully their faculties in the capacity of Medical practitioners under proper limitations. The offices of the Physicians, indeed, in some respects, are peculiary appropriate, as they would be congenial to the other sex. Poetry invests woman with the character of a "ministering angel" in the hour of sickness, and this idea almost ceases to be a figure of speech when we witness the labors of "sisters of charity" (professedly so, or not) devoted to the claims of the poor and friendless, enhanced by the sufferings of disease. Surely such philanthropic efforts should not be deprived of the vastly increased efficiency which they may derive from medical knowledge. The poetical idea of a "ministering angel," or the more prosaic character of a "sister of charity," cannot suffer depreciation, but quite the reverse, by being associated with medical education.

But, aside from purely philanthropic ends, we are inclined to think that the exercise of the duties of the medical practitioner, is by no means, an exclusive prerogative of the lords of creation, but may be claimed as belonging equally to the softer sex. The discussion of the subject would, however, lead us too deeply into the metaphysics of woman's rights, and we therefore waive it for the present.

Another inquiry is, what influence upon the character of the medical profession will be exerted by well educated female physicians? The effect, as it seems to us, will be good. We imagine that it may conduce to the diminution of quackery. Without intending any disrespect to the fair sex, we may remark, what every practitioner must have observed, that the encouragements bestowed upon the various medical delusions, and empirical schemes of the day, is, in a great degree, owing to female influence. Why it is so we will not stop to inquire. Such, at all events, is the fact. Now one result which may reasonably be expected to follow from the education of intelligent females in medical science, is the diffusion of a more rational appreciation of, and consequent respect for the medical profession, and medical knowledge, among persons of their own sex. Physicians will have fewer annoyances to suffer from the hands of ignorant nurses, and traditionary whims and notions. We have not time nor space to pursue this inquiry. Having fairly committed (without any affectation of gallantry) our first impressions in behalf of female medical education, we leave the subject for the present, to be, perhaps, resumed at some future time.

